# Supplementary material for: Impact of different visceral metastatic sites on survival in metastatic prostate cancer patients
Source: PLoS One. 2024 Sep 6;19(9):e0309941. doi: 10.1371/journal.pone.0309941 (PMC11379235; doi:10.1371/journal.pone.0309941)
Supplement: Attachment — Submitted filename: Response to Reviewers.docx [file pone.0309941.s007.docx]

Dear Reviewers:

I am deeply grateful for the time and effort you dedicated to reviewing my article. Your feedback and thoughtful questions have been incredibly valuable in refining my work. I appreciate your constructive suggestions and am committed to addressing each point you raised as follows.

Reviewer #1: 

1. In the analysis of race/ethnicity, I recommend using the terms "African Americans" and "Caucasians" instead of "Black" and "White," respectively. Additionally, please note that "Black" was incorrectly reported as "Blake" in the summary.

The “Blake” and “White” have changed to "African Americans" and "Caucasians", respectively, in the revised manuscript. Thank you for your recommendation.

2. I suggest adding the lack of information on the BRCA status of patients as a limitation.

The lack of data on BRCA mutation status of patients has been added in the limitation section. Thank you for your recommendation.

3. Additionally, the absence of data on family history of PCa should be mentioned among the limitations.

The absence of information on family history of prostate cancer has been added in the limitation section. Thank you for your recommendation.

4. The limitations should also include the absence of details regarding the modality of diagnosis and follow-up (i.e., CT scan, bone scintigraphy, PET-CT scan).

The lack of data regarding the methodology for diagnosis and follow-up has been added in the limitation section. Thank you for your recommendation.

Reviewer #2:

The lack of data regarding the methodology for diagnosis and follow-up has been added in the limitation section. Thank you for your recommendation.
